# Utility of neutrophil CD64 in distinguishing bacterial infection from inflammation in severe alcoholic hepatitis fulfilling SIRS criteria

**DOI:** 10.1038/s41598-021-99276-y

**Published:** 2021-10-05

**Authors:** Gaurav Pandey, Harshit Singh, Saurabh Chaturvedi, Manjunath Hatti, Alok Kumar, Ravi Mishra, Prabhakar Mishra, V. P. Krishna, Arun Bhadauria, Samir Mohindra, Durga Prasanna Misra, Vivek Anand Saraswat, Vikas Agarwal

**Affiliations:** 1 Gastroenterology and Hepatology, Sanjay Gandhi Postgraduate Institute of Medical Sciences, Lucknow, India; 2 Department of Clinical Immunology and Rheumatology, Sanjay Gandhi Postgraduate Institute of Medical Sciences, Raebareli Road, Lucknow, Uttar Pradesh 226014 India; 3 Department of Gastroenterology, Sanjay Gandhi Postgraduate Institute of Medical Sciences, Lucknow, Uttar Pradesh 226014 India; 4 Department of Statistics, Sanjay Gandhi Postgraduate Institute of Medical Sciences, Lucknow, India

**Keywords:** Biomarkers, Gastroenterology

## Abstract

To assess utility of neutrophilCD64 (nCD64) expression in differentiating bacterial infection from inflammation in patients with severe alcoholic hepatitis (SAH) fulfilling systemic inflammatory response syndrome criteria. Patients with SAH and infection (n = 58), SAH without infection (n = 70), and healthy controls (n = 20) were included. Neutrophil CD64 expression by flowcytometry, serum Procalcitonin (ELISA) and C-reactive protein (Nephelometry) and neutrophil–lymphocyte ratio (NLR) were studied. Percentage of neutrophils with CD64 expression (nCD64%) was significantly higher in patients with SAH and infection than in those without infection and controls [76.2% (56.9–86.5) vs. 16% (12.6–23.1) vs. 7.05% (1.4–9.5), *p* < 0.05], as was their mean fluorescence intensity [MFI; 1431 (229–1828) vs. 853 (20–968) vs. 99.5 (54.7–140.7), *p* < 0.05]. Using a cut-off of 27%, the sensitivity and specificity of nCD64% to diagnose bacterial infection was 94% and 81%, respectively, with area under curve (AUC) of 0.95. At a cut-off value of 0.261 ng/ml, the sensitivity and specificity of serum procalcitonin was 83% and 72%, respectively, with AUC of 0.86. Serum CRP, total leukocyte count, NLR had AUCs of 0.78, 0.63 and 0.64, respectively. Quantitative measurement of nCD64 can better distinguish systemic bacterial infection and inflammation in SAH as compared to traditional biomarkers.

## Introduction

Severe alcoholic hepatitis (SAH) is defined by a Maddrey’s modified discriminant function (mDF) score of 32 or more and is associated with a poor prognosis (28-day and 1-year mortality rates 30% and 50%, respectively^1–3^. Bacterial and opportunistic infections are a major cause of morbidity and mortality in patients with SAH, which may be up to 30% at 2-months^4^.The STOPAH trial had 24% incidence of severe infections in SAH^5^.The systemic inflammatory response syndrome (SIRS) is often present at admission and has an independent association with multi-organ failure and high mortality in SAH^6^. In a cohort of 162 biopsy proven SAH, Michelena et al. reported 62.1% mortality in patients who developed multi organ failure during a 90-day follow up period^6^.

Increased susceptibility to bacterial infections in SAH is due to endotoxin induced neutrophil suppression^7^ and high expression of inhibitory receptors on lymphocytes, particularly programmed cell death 1 (PD1), T-cell immunoglobulin and mucin domain–containing protein 3 (TIM3) and their ligands PD ligand 1 (PDL-1) and galectin-9, respectively^8^. Higher expression of inhibitory receptors on lymphocytes and neutrophil dysfunction may result in immune paralysis and increased susceptibility to infections^9^.The phenomenon of T cell exhaustion in SAH and impaired adaptive and innate immunity in acute AH have been well documented^8,10^.

Besides supportive care and nutritional support, glucocorticoid is the most widely used therapy in AH; however, glucocorticoids use is contraindicated in presence of severe infection. Patients with SAH are particularly vulnerable to bacterial infections, hence differentiating SIRS(without infection) from systemic infection is vitally important before initiating glucocorticoid therapy in them. However, this remains problematic in routine clinical practice since fever, raised total leukocyte count (TLC) and C-reactive protein (CRP) in SAH may be due to SAH and SIRS per se or due to bacterial infection, thus highlighting the need for biomarker(s) that can reliably differentiate between the two. Detecting infection in pre-existing inflammatory milieu has remained an unresolved conundrum till date^11^. Common screening laboratory tests employed to diagnose infections, such as TLC, presence of immature forms in peripheral smear, CRP and erythrocyte sedimentation rate (ESR), have poor specificity^12^. Culture results are often viewed as confirmatory, but in practice culture yields are low and results are often available too late to be of use in making immediate treatment decisions. In a condition where 30-day mortality is very high, delay of even a few hours may prove to be detrimental to the outcome.

In an observational study, Ajmani et al. found that surface expression of CD64 on neutrophils could be useful for differentiating between infective and non-infective inflammation^13^. The Fc receptors are members of the immunoglobulin supergene family found on white blood cells, where they function to integrate responses involving both the innate and acquired immune systems. CD64 is the Fc receptor that binds with monomeric IgG type antibodies with high affinity. Due to its high avidity for IgG, CD64 (FCγR1) plays an important role in phagocytosis, explaining the functional relevance of inducing CD64 on neutrophils^11^. During an infection, CD64 expression on neutrophils is induced by interferon-γ (IFN‐γ), granulocyte monocyte colony stimulating factor (GM‐CSF) and other mediators released by macrophages in response to pathogen associated molecular patterns (PAMPs)^11^. Normally, CD64 is present on the surface of only a few circulating polymorphonuclear leukocytes (PMN), but, within 4  to 6 hours of an infection, neutrophil CD64 expression (nCD64) rapidly increases in response to microbial wall components, complement split products and some pro-inflammatory cytokines^14–16^. Previous studies have found nCD64 expression to be a useful marker for systemic infection^17,18^. A meta-analysis found percentage of neutrophils expressing nCD64 (nCD64%) to be a good biomarker for early diagnosis of systemic infection with area under receiver operating characteristics curve of 0.95(Q* = 0.89)^19^.

Although nCD64 has been found to be a useful marker of systemic infection, it remains unclear whether nCD64 can distinguish infection from active inflammation in patients with inflammatory diseases associated with activation of the immune system and increased pro-inflammatory cytokines milieu, who are also susceptible to infections. In the present study, we have evaluated the clinical usefulness of quantitative nCD64 measurements to differentiate between systemic bacterial infection and active inflammation in patients with SAH.

## Results

**Figure 1:**
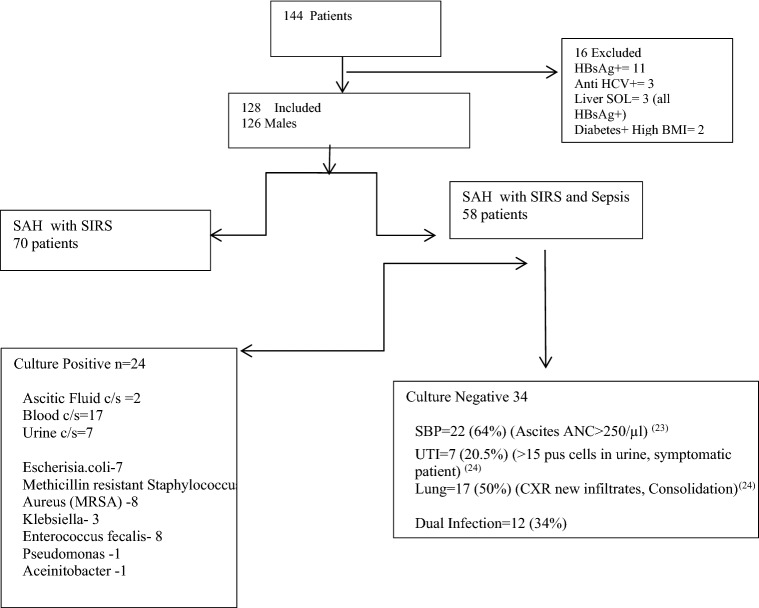
Flow diagram showing enrolment of severe alcoholic hepatitis patients recruited in the study.

Sixteen of 144 patients enrolled were excluded as they were HBsAg positive (n = 11), anti-HCV positive (n = 3) or had a space occupying lesion in the liver (n = 2). Finally, 128 patients (126 males and 2 females), and 20 healthy controls were included (Fig. 1).

Mean age of the patients was 43.8 ± 8.5 (range 27–70 years) and the mean mDF score was 105.4 ± 54.2. None of the patients were receiving glucocorticoids at the time of enrolment in the study. Infection group included 58 patients with proven or probable infection while non-infection group included 70 patients with inflammation who did not have infection. When baseline characteristics of patients in the two groups were compared (Table 1), patients in Infection group had significantly higher international normalized ratio (*p* < 0.001) and MELD score (*p* < 0.001) compared with patients in non-infection group. Fever at presentation was observed in 73% of patients in Infection group and 42% in non-infection group (*p* < 0.0005). Twenty-four patients in Infection group had culture proven infection (18 blood cultures, 8 urine cultures and 2 ascitic fluid cultures, 4 patients had dual source of infections), while 34 had probable infection [22 Spontaneous bacterial peritonitis^20^ (SBP)], 17 lung infections, 7 urinary tract infection^21^ and 12 patients had dual source). Predominant sites of infections in the probable infection were ascitic fluid (SBP; 54%), urinary tract (23%), lower respiratory infection (15%), and cutaneous thrombophlebitis (3%). More than a quarter (29%) had more than 1 site of infection. The organisms isolated included *E.coli (n* = *7), methicillin resistant Staphylococcus aureus (n* = *8), Klebsiella (n* = *3), Enterococcus faecalis (n* = *8), Acinetobacter (n* = *1) and Pseudomonas (n* = *1).* Four patients had dual organism positivity from two different sites.

**Table 1:** Baseline characteristics of patients in severe alcoholic hepatitis with Infection vs non-infection group.

Variable´s	Infection group (n = 58)	Non-infection group (n = 70)	*P* -value
Age	41.08 ± 6.73	46.83 ± 9.20	< 0.01
INR	2.95 ± 1.6	1.9 ± 0.62	< 0.001
Serum Bilirubin (mg/dl)	14.2 ± 11.2	12.3 ± 2.7	0.376
Serum Albumin (g/dl)	2.5 ± 1.3	2.7 ± 1.6	0.446
S Na^+^ (meq/dl)	128 ± 11.2	132 ± 10.6	0.041
CTP	11.2 ± 2.9	10.3 ± 1.9	0.037
MELD	34.4 ± 5.4	28.5 ± 5	< 0.001
mDF	142 ± 31	72.5 ± 24	< 0.001
Serum Creatinine (mg/dl)	1.5 ± 0.9	1.08 ± 0.78	0.006
Number of OF	2 (1–4)	1 (1–4)	ns
ACLF Grade	2 (1–3)	1 (1–2)	ns
TLC (× 10^3^/µl)	14.4 ± 7.6	14.1 ± 7.5	0.76
Heart rate per minute	93 ± 12.7	91.8 ± 9.3	0.87
Respiratory Rate per minute	22 (16–30)	19 (16–26)	ns

Data is presented as mean ± SD. Independent samples t test used, *p* < 0.05 significant.

*INR* international normalized ratio, *CTP* Child-Turcotte-Pugh Score, *MELD* Model for End Stage Liver Disease, *mDF* Maddrey’s Discriminant Function, *OF* organ failure, *ACLF* acute on chronic liver failure, *TLC* total leukocyte count.

### Neutrophil CD64 expression

**Figure 2:**
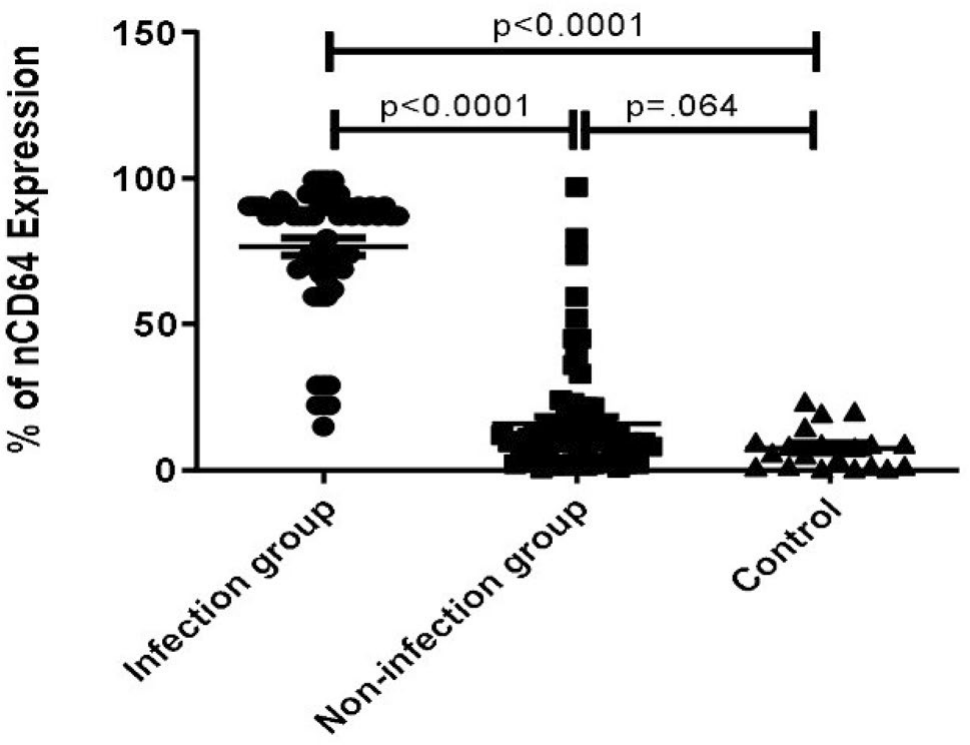
Scatter plot showing distribution of neutrophil CD64 expression between severe alcoholic hepatitis patients with and without infection vs healthy controls.

**Figure 3:**
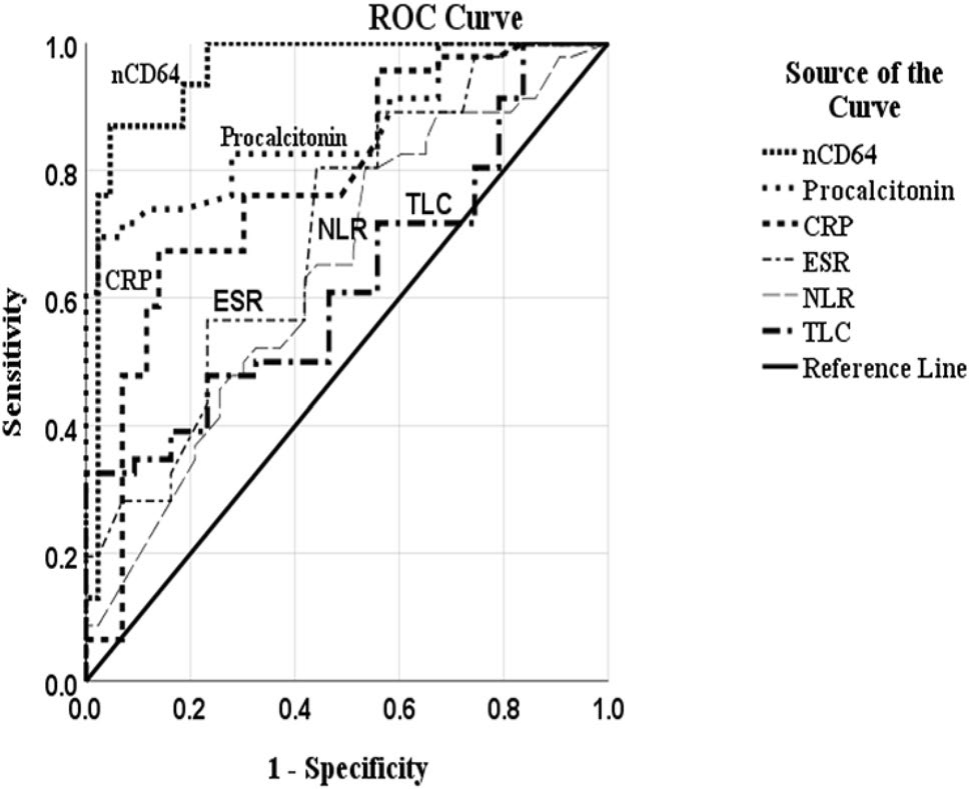
Receiver operating characteristic curves of neutrophil CD64 expression, serum procalcitonin, Neutrophil:lymphocyte ratio, C-reactive protein, erythrocyte sedimentation rate and total leukocyte count in patients of severe alcoholic hepatitis with infection and non-infection groups.

nCD64% was significantly higher in Infection group [76.2% (range 56.9–86.5)] as compared to non-infection group [16% (12.6–23.1)] and healthy controls [nCD64 7.05% (range 1.4–9.5), *p* < 0.05] (Fig. 2, Table 2). nCD64% was not significantly different between healthy controls and non-infection group patients. Similarly, MFI was significantly higher in Infection group [1431 (229–1828)] when compared with non-infection group [853 (20–968)] and healthy controls. With receiver operator characteristics (ROC) curve analysis, nCD64 cut-off value of 27% was 93% sensitive and 85.7% specific for detection of bacterial infection from patients without infection with AUC of 0.95 (Tables 3, 4; Fig. 3). There was no correlation between duration of fever and nCD64%. The positive and negative predictive values of nCD64% were 84% and 94%, respectively which were higher than any other biomarkers; serum procalcitonin, NLR (Neutrophil–Lymphocyte Ratio), CRP and ESR. We found a weak positive correlation between nCD64% and MELD Score [*r* :0.41 (*p* < 0.01)].

**Table 2:** Biomarkers to differentiate between infection vs inflammation in severe alcoholic hepatitis.

Groups	N	Mean	SD	*P* value
**nCD64%**				
Infection	58	76.66	22.60	< 0.001
Non- Infection	70	16.02	18.96	
**MFI of nCD64**				
Infection	58	1431	359	< 0.001
Non- Infection	70	853	257	
**CRP (mg/dl)**				
Infection	58	6.20	4.84	< 0.001
Non- Infection	70	3.11	3.57	
**ESR (mm 1st hour)**				
Infection	58	91.18	37.59	< 0.001
Non- Infection	70	63.06	41.46	
**Serum Procalcitonin (ng/ml)**				
Infection	58	0.438	0.240	< 0.001
Non- Infection	70	0.185	0.128	
**TLC (/µl)**				
Infection	58	11,108.19	6911.99	0.001
Non- Infection	70	7402.50	4166.63	

Data is presented as mean ± SD. Independent samples t test used. #Presented in median (interquartile range) and compared by Mann Whitney U test used. *p* < 0.05 significant.

*nCD64%* Neutrophils CD64 expression in percentage, *MFI* Mean fluorescence intensity, *CRP* C-reactive protein, *ESR* erythrocyte sedimentation rate, *TLC* total leukocyte count.

**Table 3:** Diagnostic accuracy of the various biomarkers (N = 128).

SR.no.	Biomarker	Cut-off	AUC	Sensitivity (%)	Specificity (%)	Positive predictive value	Negative predictive value
1	nCD64%	26.5	0.95	93 (0.84–0.97)	85.7 (0.76–0.92)	0.844 (0.74–0.91)	0.94 (0.85–0.97)
2	Serum Procalcitonin (ng/ml)	0.261	0.86	74.1 (0.61–0.84)	70 (0.59–0.80)	0.67 (0.55–0.77)	0.77 (0.65–0.85)
3	CRP (mg/dl)	5.30	0.78	70.7 (0.58–0.80)	65.7 (0.54–0.76)	0.63 (0.51–0.74)	0.73 (0.61–0.82)
4	ESR (mm 1st hour)	94.5	0.71	46.6 (0.34–0.59)	82.9 (0.72–0.90)	0.69 (0.54–0.81)	0.65 (0.55–0.74)
5	NLR	4.16	0.64	60.3 (0.48–0.72)	47.1 (0.36–0.59)	0.49 (0.37–0.60)	0.59 (0.460.71)
6	TLC (/µl)	9200	0.63	56.9 (0.44–0.69)	66 (0.54 0.76)	0.58 (0.45–0.70)	0.63 (0.53–0.75)

Area under curve (AUC) was estimated using ROC curve. *p* < 0.05 significant.

Results are expressed as Estimate (95% Confidence intervals).

*nCD64%* Neutrophils CD64 expression in percentage, *CRP* C-reactive protein, *ESR* erythrocyte sedimentation rate, *NLR* neutrophil: lymphocyte ratio, *TLC* total leukocyte count.

**Table 4:** Cut off values of the Inflammatory variables using ROC Curve (N = 128).

Test result Variable(s)	Positive if greater than or equal to	Sensitivity	False negative rate	1—Specificity (False positive rate)	Specificity
nCD64%	22.05	1.00	0.00	0.23	0.77
	26.50	0.94	0.06	0.19	0.81
	59.47	0.87	0.13	0.05	0.95
	73.80	0.76	0.24	0.02	0.98
CRP (mg/dl)	0.33	1.00	0.00	0.84	0.16
	0.66	0.98	0.02	0.67	0.33
	1.71	0.96	0.04	0.56	0.44
	1.84	0.93	0.07	0.56	0.44
	2.61	0.76	0.24	0.30	0.70
	5.30	0.67	0.33	0.14	0.86
	5.52	0.59	0.41	0.12	0.88
ESR (mm 1st hour)	15.00	1.00	0.00	0.84	0.16
	28.75	0.98	0.02	0.74	0.26
	47.00	0.89	0.11	0.56	0.44
	65.00	0.80	0.20	0.44	0.54
	94.50	0.57	0.43	0.23	0.77
TLC (/µl)	2300	1.00	0.00	0.89	0.11
	2950	0.91	0.09	0.83	0.17
	3450	0.80	0.20	0.78	0.22
	5850	0.72	0.28	0.61	0.39
	6750	0.61	0.39	0.56	0.44
	9200	0.50	0.50	0.39	0.61
Serum Procalcitonin (ng/ml)	0.063	1.00	0.00	0.67	0.33
	0.173	0.91	0.09	0.58	0.42
	0.195	0.85	0.15	0.56	0.44
	0.261	0.83	0.17	0.28	0.72
	0.346	0.74	0.26	0.12	0.88
	0.375	0.72	0.28	0.07	0.93

When Values are increasing, chances of the infections are also increasing i. e. Higher values Indicate more positive (infections).

*nCD64%* Neutrophils CD64 expression in percentage, *CRP* C-reactive protein, *ESR* erythrocyte sedimentation rate, *TLC* total leukocyte count.

### Serum procalcitonin

Serum procalcitonin was higher in Infection group patients as compared to those in non-infection group. At a cut off value of 0.261 ng/ml it had a sensitivity and specificity of 83% and 72%, respectively to differentiate between the two, with an area under the curve of 0.85 (Tables 3, 4, Fig. 3). Other biomarkers; CRP, ESR, TLC and NLR had lower sensitivity and specificity scores (Tables 3, 4).

### Combining tests

If either a high nCD64% or serum procalcitonin was taken to indicate infection, then sensitivity increased to 98.3%, however, specificity was reduced to 62%. Combination of nCD64% and CRP had a sensitivity of 98%, but low specificity 55% (Supplementary Table 1, Supplementary Table 2).

#### Validation cohort

We performed nCD64% in 38 patients with acute on chronic liver failure (ACLF) of similar severity with (n = 27) and without (n = 11) infections. The mean nCD64% was 86.5% in infection vs 16.6% in non-infection patients. However, this was a mixed cohort of ACLF patients.

## Discussion

It is challenging to differentiate acute inflammation from systemic bacterial infection, particularly in patients requiring immunosuppressive medications for treatment. Bacterial infections are the most feared because they can evolve very rapidly and can be life-threatening within hours to a few days. We observed higher nCD64% in patients of SAH with infection compared to SAH without infection. It had better area under the curve than serum procalcitonin, CRP, ESR, NLR and TLC. To the best of our knowledge, this is the first study in which nCD64% was used to differentiate infection from SIRS without infection in SAH.

Bakke and colleagues reported for the first-time that quantitative measurement of nCD64 held promise for detection of systemic infection^22^. In an earlier study of systemic inflammatory diseases [systemic lupus erythematosus (SLE) and anti-neutrophil cytoplasmic antibody associated vasculitis], Ajmani et al. reported a sensitivity and specificity of 85% and 84%, respectively at a nCD64% cut-off of 30%, to differentiate between infection and inflammation^13^. In another study in active autoimmune inflammatory conditions and vasculitis, a high sensitivity (85%) and specificity (91%) for distinguishing between systemic infection and active inflammation was reported^23^. Similarly, in another study conducted on patients with SLE and rheumatoid arthritis, diseases which require immunosuppressive therapy, nCD64%was observed to be significantly higher in patients with infection as opposed to patients without infection and had a sensitivity of 94.4% with specificity of 89.9% for differentiating infection from disease activity^24^. Results of the present study are in accord with these data.

Severe AH is an intensely inflammatory condition on one hand and is complicated by infections in almost one-half of patients on the other. This creates a major dilemma for the clinician about the timing of offering glucocorticoid therapy, before or after antibiotic therapy. Early introduction of antibiotics at the time of admission even in absence of infection has not been reported to reduce mortality or incidence of subsequent infections in AH^21^. Both SIRS and infections are major causes of mortality and require diverging therapeutic strategies. High dose glucocorticoids may reduce short term mortality in SIRS but may predispose towards or worsen existing infection. In such a scenario, percentage nCD64 expression may provide guidance in choosing the right treatment. Additionally, nCD64 expression is not affected by steroid therapy, making it even more suitable for further evaluation for early detection of bacterial infection in patients with SAH receiving glucocorticoids^25^.

Recently, NLR has been reported to be a better diagnostic marker to detect bacterial infection in hospitalized patients with fever^26^, additionally day-4 NLR has prognostic value for 90-day mortality in patients with SAH^27,28^. STOPAH trial reported NLR data for 789 patients. NLR was associated with acute kidney injury and infection. In a cohort of patients with SAH, those with baseline NLR score between 5 and 8 were more likely to respond to glucocorticoids^28^.

For a long time, serum procalcitonin has been regarded as the best diagnostic test available for systemic infection. However, serum procalcitonin has been reported to be elevated in patients with severe liver dysfunction without infection, limiting its diagnostic utility for infection in patients with SAH^29^. In a recent meta-analysis, the sensitivity and specificity of procalcitonin (88% and 81%) was reported to be higher than that of CRP (75% and 67%)^30^. Our study demonstrated that nCD64% was more sensitive and specific than serum procalcitonin, CRP, NLR, ESR or TLC in the detection of bacterial infections in SAH patients. The AUC in our study for nCD64% at 0.95 was consistent with other studies and meta-analysis^19^ on the same marker for bacterial infection in other disease conditions.

Ours being a tertiary care referral center receive patients in advanced stage of the disease with high mDF and MELD scores and are generally initiated on antibiotics at peripheral centers so the number of culture positive patients was low. However, all the patients categorized in our study as probable infection had infections based on a composite of clinician’s judgement and laboratory investigations despite negative microbial cultures.

There are few limitations of our study; small sample size of the validation cohort and it being a mixed cohort of ACLF patients rather than SAH patients. The mean mDF score of 105 was extremely high in our study therefore studies with larger number of patients and mDF > 32 but < 100 and MELD > 20 should be done to validate it in future studies. Other limitation is the lacking assessment of other biomarkers; interferon-γ, lipopolysaccharides and other cytokines in plasma of these patients. Majority of patients in the present study were males, probably due to the socio-cultural milieu of our population. Due to cross-sectional design of the study, we did not assess the correlation between the nCD64% and the outcome. Despite limitations, taking into consideration the fact that nCD64% has performed almost equally well in other systemic inflammatory diseases like ANCA associated vasculitis and SLE and the data obtained from the current study, nCD64% ability to distinguish bacterial infection from SIRS makes a strong case for its utility in SAH patients as well.

In conclusion, we propose that nCD64% expression may be a useful tool to differentiate bacterial infection from SIRS in patients with SAH. It is a simple test with a short turn-around time (2–4 h) and has a cost comparable with CRP and pro-calcitonin (about 8 USD per assay in our laboratory). In a setting of SAH where 30-day mortality is very high and definite evidence of infection in form of blood and other body fluid cultures may take 3–5 days, a short turn-around time marker such as nCD64% may facilitate timely initiation of appropriate treatment and is likely to impact prognosis. These characteristics make it an attractive test to incorporate into routine clinical practice for the management of patients with SAH.

## Material and methods

This prospective, cross sectional, observational descriptive study was conducted in the Department of Gastroenterology, Sanjay Gandhi Postgraduate Institute of Medical Sciences, Lucknow, India from March 2017 to June 2018.

Alcoholic hepatitis was diagnosed as per National Institute on Alcohol Abuse and Alcoholism (NIAAA), Alcoholic Hepatitis Consortia criteria^31^ after exclusion of other liver diseases in a patient with a long history of heavy alcohol use (typically > 100 g per day for more than 5 years) with recent and rapid development or worsening of jaundice, liver related complications and serum bilirubin of > 3 mg/dl, along with elevation in aspartate aminotransferase (AST) and alanine aminotransferase (ALT) levels to more than 1.5 times the upper limit of normal but less than 400 U/l, with AST/ALT ratio of > 1.5 and with persistent alcohol use until 8 weeks before onset of symptoms^31^. Severe AH was defined as a mDF score of 32 or more^32^. The definition of SIRS used was presence of ≥ 2 of the following: (1) temperature > 38 °C or < 36 °C (2) Respiratory Rate > 20/min (3) heart rate > 90/min (4) White blood cell count > 12,000/µl/ < 4000/ or > 10% bands^33^**.** Liver biopsy was carried out when there was a doubt in diagnosis due to overlapping features in patients with decompensated cirrhosis with ongoing or recent alcohol abuse and severe alcoholic hepatitis.

Sepsis by definition is systemic inflammatory response (SIRS) due to infection- microbial etiology (proven or suspected)^33^. In our study, SIRS and any one of the following mentioned below was used to diagnose Sepsis: (1) positive blood/urine/ascitic fluid/sputum culture, (2) spontaneous bacterial peritonitis (SBP; as defined by infection of the ascitic fluid, as evidenced by an ascitic fluid absolute polymorphonuclear leukocyte (PMN) count of at least 250 cells/µl (0.25 × 10^9^/L), with or without a positive ascitic fluid culture, in the absence of an intra-abdominal surgically treatable source of infection^20^ (3) lower respiratory tract infections with new pulmonary infiltrates in the presence of respiratory symptom/s (cough, sputum production, dyspnea, pleuritic pain) with rales or crepitation on auscultation or one sign of infection (core body temperature > 38 °C or < 36 °C, shivering or leukocyte count > 10,000/mm^3^ or < 4000/mm^3^) in the absence of antibiotics or new infiltrates or consolidation/lung abscess demonstrable on radiograph/ computed tomography of chest, (4) evidence of extra hepatic biliary obstruction or hepatic abscess on abdomen ultrasound (5) skin infections; fever with cellulitis, abscess, discharging pus from the skin lesion (6) urinary tract infection (UTI) being diagnosed as positive urine dipstick result for leukocytes or nitrites, or urine WBC > 15/high-power field with either positive urine gram stain or culture in a symptomatic patient^21^.

### Patient

All consecutive patients suffering from SAH were evaluated at Gastroenterology department, and those with SIRS or SIRS with infection were prospectively recruited in the study after obtaining informed consent. Study was approved by Institutional ethics committee, Sanjay Gandhi Postgraduate Institute of Medical Sciences (A-18-PGI-IMP-75-2017) and was performed in accordance with the Declaration of Helsinki. All laboratory experiments were performed in accordance with relevant guidelines and regulations.

#### Inclusion criteria

Patients aged 18 years or more, suffering from SAH according to clinical, biochemical, imaging and liver tissue pathology criteria with mDF score of more than 32, model for end stage liver disease (MELD) score > 20 and suspected to have either SIRS or SIRS with infection were included in this study.

#### Exclusion criteria

Patients with absolute neutrophil count less than 1500 per microliter (μl), those who had received granulocyte-colony stimulating factor (G-CSF) therapy, who were suspected to have mycobacterial or fungal infection who were on immunosuppressants (other than glucocorticoids) or chemotherapeutic agents, who had been found to have hepatocellular carcinoma or other malignancy, who had sustained trauma or undergone surgery, or had suffered from some vital disease other than liver disease within 3 months of study entry were excluded^6^.

Patients were divided into two groups, namely those with SAH and SIRS with infection (Infection Group, 58 patients) and those with SAH with SIRS (Non-infection Group, 70 patients). Infection was considered proven when a pathogen was detected by microbial culture techniques. Infection was considered probable in patients with a composite of suspicion of infection in the form of fever and raised TLC (> 10,000/mm^3^) or Leukopenia (< 4000/mm^3^) plus any one of the following: Spontaneous bacterial peritonitis, Radiological findings suggesting new infiltrates, consolidation or lung abscess on chest radiograph or computerized tomography (CT), Pus discharge from skin lesions, Cellulitis which were culture negative due to prior antibiotic therapy initiated prior to referral to our center. Infection was considered unlikely if workup to rule out infection, including chest radiograph, urine examination, ascitic fluid evaluation, and appropriate body fluid cultures turned out to be negative. Patients with proven and probable infection were analyzed together and compared with those with inflammation but no infection. Pending cultures all patients fulfilling the criteria of SAH and SIRS were initiated on antibiotics.

Six milliliters of venous blood (3 ml each in a plain vial and in ethylene diamine tetra acetic acid (EDTA) was collected from each patient and healthy controls. Laboratory personnel carrying out the tests were unaware to the patients’ clinical disease status.

### Neutrophil CD64 expression

nCD64 was analyzed via flow-cytometry as previously described^13^. Briefly, 1 ml EDTA blood was incubated for 30 min at room temperature with 20 µl phycoerythrin (PE) conjugated human anti-CD64 antibody (BD Pharmingen CA, USA) and 20 µl PE-conjugated matched-isotype control antibody, followed by lysis of RBCs and two steps of washing with phosphate buffer saline. Results are expressed as the percentage of neutrophils expressing CD64 (nCD64%) and as mean fluorescence intensity (MFI).

### Serum procalcitonin

Blood samples were allowed to clot for 45 min, then centrifuged at 1500 rpm for 10 min and serum was aliquoted and stored at − 80 °C until analysis. Procalcitonin levels were measured in duplicate after a single freeze–thaw cycle in batched assays by sandwich enzyme-linked immunosorbent assay (ELISA), R&D systems (catalog No DY8350-05), as per manufacturer’s protocol.

### Lab parameters

C-reactive protein (CRP) in the serum was measured by nephelometry. ESR was measured by Westergren method.

### Statistical analysis

Normality of the continuous variables was assessed and considered normally distributed when Z score of the skewness was ± 3.29. Descriptive statistics of the continuous variables were presented as mean ± standard deviation (S.D.) or median (interquartile range) whereas categorical data were presented in frequency (%). Independent samples t test was used to compare the means between two groups whereas Mann Whitney U test used to compare the non-normal continuous variables between two groups. Receiver operating characteristics (ROC) curve was used to estimate the diagnostic accuracy (area under curve “AUC”, sensitivity, specificity) of the inflammatory variables with corresponding significance levels. AUROC cut-offs were chosen by taking the optimum balance between the sensitivity and specificity. The value was provided as given in the output of the software used for statistical analysis (SPSSv23). To compute the sensitivity and specificity of the two combined variables, either variable (based on identified cutoff value of nCD64% of 26.4 and Pro-calcitonin of 0.261) present was considered positive (infection present) and it was compared with clinical evidence of infection (both probable and confirmed infection) as the gold standard. A *p* value < 0.05 was considered statistically significant. Statistical package for social sciences, version-23 (SPSS-23, IBM, Chicago, USA) and MedCalc statistical software was used for data analysis.

## Supplementary Information

Supplementary Information.

## Abbreviations

ELISA: Enzyme-linked immunosorbent assay
ESR: Erythrocyte sedimentation rate
MAS: Macrophage activation syndrome
MFI: Mean fluorescence intensity
PBS: Phosphate buffer saline
PMN: Polymorphonuclear leukocytes
