# Supplementary material for: Utility of neutrophil CD64 in distinguishing bacterial infection from inflammation in severe alcoholic hepatitis fulfilling SIRS criteria
Source: Sci Rep. 2021 Oct 5;11:19726. doi: 10.1038/s41598-021-99276-y (PMC8492738; doi:10.1038/s41598-021-99276-y)
Supplement: Supplementary file 1 — Supplementary Information. [file 41598_2021_99276_MOESM1_ESM.docx]

| Supplementary Table 1: Receiver operating characteristics curve value on combining nCD64% with serum Procalcitonin | | | | | |
| --- | --- | --- | --- | --- | --- |
|  | | | Observed Outcomes | | Total |
|  |  |  | Infection | No Infection |  |
| Combined results  [Serum procalcitonin and nCD64] | Infection | Count | 57 | 27 | 84 |
|  |  | % within row | 67.9% | 32.1% | 100.0% |
|  |  | % within column | 98.3% | 38.0% | 65.1% |
|  | Non- Infection | Count | 1 | 43 | 44 |
|  |  | % within row | 2.2% | 97.8% | 100.0% |
|  |  | % within column | 1.7% | 62.0% | 34.9% |
| Total | | Count | 58 | 70 | 128 |
|  |  | % within row | 45.0% | 55.0% | 100.0% |
|  |  | % within column | 100.0% | 100.0% | 100.0% |
| Sensitivity =98.3%, False negative rate = 1.7%  Specificity =62%, False negative rate = 38.0%  nCD64%; Neutrophils CD64 expression in percentage | | | | | |

| Supplementary Table 2: ROC value on combining nCD64% with serum CRP | | | | | |
| --- | --- | --- | --- | --- | --- |
|  | | | Observed Outcomes | | Total |
|  |  |  | Infection | No Infection |  |
| Combined Results  [CRP and nCD64] | Infection | Count | 57 | 31 | 88 |
|  |  | % within row | 64.0% | 36.0% | 100.0% |
|  |  | % within column | 98.3% | 45.1% | 69.0% |
|  | Non- Infection | Count | 1 | 39 | 40 |
|  |  | % within row | 2.5% | 97.5% | 100.0% |
|  |  | % within column | 1.7% | 54.9% | 31.0% |
| Total | | Count | 58 | 71 | 128 |
|  |  | % within row | 45.0% | 55.0% | 100.0% |
|  |  | % within column | 100.0% | 100.0% | 100.0% |
| Sensitivity =98.3%, False negative rate = 1.7%  Specificity =54.9%, False negative rate = 45.1%  nCD64%; Neutrophils CD64 expression in percentage, CRP; C-reactive protein | | | | | |

STARD Checklist

|  | **Section & Topic** | **No** | **Item** | **Reported on page #** |
| --- | --- | --- | --- | --- |
|  |  |  |  |  |
|  | **TITLE OR ABSTRACT** |  |  |  |
|  |  | **1** | Identification as a study of diagnostic accuracy using at least one measure of accuracy  (such as sensitivity, specificity, predictive values, or AUC) | 1 |
|  | **ABSTRACT** |  |  |  |
|  |  | **2** | Structured summary of study design, methods, results, and conclusions  (for specific guidance, see STARD for Abstracts) | 4 |
|  | **INTRODUCTION** |  |  |  |
|  |  | **3** | Scientific and clinical background, including the intended use and clinical role of the index test | 5 |
|  |  | **4** | Study objectives and hypotheses | 6 |
|  | **METHODS** |  |  |  |
|  | *Study design* | **5** | Whether data collection was planned before the index test and reference standard  were performed (prospective study) or after (retrospective study) | 6 |
|  | *Participants* | **6** | Eligibility criteria | 7 |
|  |  | **7** | On what basis potentially eligible participants were identified  (such as symptoms, results from previous tests, inclusion in registry) | 7 |
|  |  | **8** | Where and when potentially eligible participants were identified (setting, location and dates) | 7 |
|  |  | **9** | Whether participants formed a consecutive, random or convenience series | 7 |
|  | *Test methods* | **10a** | Index test, in sufficient detail to allow replication | 8 |
|  |  | **10b** | Reference standard, in sufficient detail to allow replication | 8 |
|  |  | **11** | Rationale for choosing the reference standard (if alternatives exist) | 8 |
|  |  | **12a** | Definition of and rationale for test positivity cut-offs or result categories  of the index test, distinguishing pre-specified from exploratory | 11 |
|  |  | **12b** | Definition of and rationale for test positivity cut-offs or result categories  of the reference standard, distinguishing pre-specified from exploratory | 11 |
|  |  | **13a** | Whether clinical information and reference standard results were available  to the performers/readers of the index test | 8 |
|  |  | **13b** | Whether clinical information and index test results were available  to the assessors of the reference standard | - |
|  | *Analysis* | **14** | Methods for estimating or comparing measures of diagnostic accuracy | 9 |
|  |  | **15** | How indeterminate index test or reference standard results were handled | - |
|  |  | **16** | How missing data on the index test and reference standard were handled | Not applicable |
|  |  | **17** | Any analyses of variability in diagnostic accuracy, distinguishing pre-specified from exploratory | - |
|  |  | **18** | Intended sample size and how it was determined | 9 |
|  | **RESULTS** |  |  |  |
|  | *Participants* | **19** | Flow of participants, using a diagram | 10 |
|  |  | **20** | Baseline demographic and clinical characteristics of participants | 18 |
|  |  | **21a** | Distribution of severity of disease in those with the target condition | 18 |
|  |  | **21b** | Distribution of alternative diagnoses in those without the target condition | - |
|  |  | **22** | Time interval and any clinical interventions between index test and reference standard | - |
|  | *Test results* | **23** | Cross tabulation of the index test results (or their distribution)  by the results of the reference standard | 19 |
|  |  | **24** | Estimates of diagnostic accuracy and their precision (such as 95% confidence intervals) | 20 |
|  |  | **25** | Any adverse events from performing the index test or the reference standard | - |
|  | **DISCUSSION** |  |  |  |
|  |  | **26** | Study limitations, including sources of potential bias, statistical uncertainty, and generalisability | 12 |
|  |  | **27** | Implications for practice, including the intended use and clinical role of the index test | 13 |
|  | **OTHER INFORMATION** |  |  |  |
|  |  | **28** | Registration number and name of registry | - |
|  |  | **29** | Where the full study protocol can be accessed | - |
|  |  | **30** | Sources of funding and other support; role of funders | 13 |
|  |  |  |  |  |
